# The correlation between serum lipid profile with carotid intima-media thickness and plaque

**DOI:** 10.1186/1471-2261-14-181

**Published:** 2014-12-09

**Authors:** Caie Yang, Zhiqiang Sun, Yunpeng Li, Junping Ai, Qiyu Sun, Yaping Tian

**Affiliations:** Department of Clinical Biochemistry, Chinese PLA General Hospital, No. 28 Fuxing Road, Beijing, 100853 China; Department of Clinical Laboratory, Chinese PLA 309 Hospital, No. 17 Heishanhu Road, Beijing, 100091 China

**Keywords:** Subclinical atherosclerosis, Lipid ratio, Intima-media thickness, Plaque

## Abstract

**Background:**

It is indicated that non-HDL cholesterol and lipid ratios, including total/HDL cholesterol and LDL/HDL cholesterol ratios, are risk indicators with greater predictive value for coronary atherosclerotic progression or regression compared with conventional lipid profile. However, there have been few reports about the correlation between serum lipid profile with carotid intima-media thickness (IMT) and plaque in Chinese general people.

**Methods:**

We examined 402 subjects without apparent diseases in a cross-sectional study (mean age 50.16 years; 36.07% female). Demographics, anthropometrics, and laboratory data were collected. The presence of carotid plaque and intima-media thickness were evaluated by ultrasonography.

**Results:**

Univariate correlations showed carotid IMT was correlated with LDL-C (r = 0.137, *p* = 0.009), non-LDL-C levels (r = 0.140, *p* = 0.008) and LDL-C/HDL-C ratio (r = 0.169, *p* = 0.001). After adjustment for potential covariates, LDL-C/HDL-C ratio (β = 0.132, *p* < 0.001) were independent variables that interacted on carotid IMT. Other risk factors including age and systolic blood pressure were independently associated with carotid IMT. LDL-C levels, non-HDL-C levels, TC/HDL-C and LDL-C/HDL-C ratios were significantly higher, but HDL-C levels were significantly lower in subjects with carotid plaque than those without it. The subsequent multiple logistic regression analysis showed that LDL-C (OR; 1.325, 95% CI; 1.046-1.821, *p* = 0.033) and HDL-C levels (OR; 0.093, 95% CI; 0.038-0.227, *p* < 0.001) were significantly associated with the presence of carotid plaque after adjustment of age. Furthermore, LDL-C combined with HDL-C levels showed the highest area under the curve (0.788, 95% CI; 0.740–0.837, *p* < 0.001).

**Conclusions:**

Serum LDL-C/HDL-C ratio represents as an independent index associated with increased carotid IMT and LDL-C combined with HDL-C levels may be useful markers for predicting the presence of carotid plaque in the Chinese general population.

## Background

Atherosclerosis is the primary cause of mortality and morbidity in cardiovascular disease (CVD) [1]. Dyslipidemia is the most important risk factor for atherosclerosis and contributes to increased risk to develop CVD. Previous studies have demonstrated that low-density lipoprotein (LDL) is the primary atherogenic lipoprotein [2, 3] and that high-density lipoprotein (HDL) is the predominant anti-atherosclerotic lipoprotein [4]. Therefore, measurements of total cholesterol (TC), HDL cholesterol (HDL-C), and LDL cholesterol (LDL-C) are widely recommended [5]. Recent data suggested that non-HDL cholesterol (non-HDL-C) is a better parameter for assessing CVD risk rather than TC and HDL-C [6–9]. Also, some studies suggested that lipid ratios, including TC/HDL-C and LDL-C/HDL-C ratios are risk factors with better predictive value for coronary atherosclerotic progression or regression than each lipid parameter used independently [10–14]. However, data comparing the role of non-HDL-C and these lipid ratios with conventional lipid profile in early-stage atherosclerotic changes are limit.

Atherosclerosis develops silently over decades before symptoms eventually occur. Carotid ultrasonography can be used to noninvasively identify early-stage atherosclerotic changes in arterial wall. Measurements of intima-media thickness (IMT) and detection of plaque formation have been used as early and sensitive indicators for early-stage atherosclerosis [15, 16].

In the present study, we investigated associations between conventional lipid parameters and lipid ratios with carotid IMT and plaque in participants of a health checkup from the general population.

## Methods

### Study population enrollment

Study subjects were randomly recruited from individuals who visited the Health Medical Center of PLA General Hospital for a health checkup from January 2014 to May 2014. All participants underwent a routine clinical examination including physical examination, blood biochemical examination and carotid ultrasonography. Those who had history of CVD, diagnosed atherosclerosis (carotid IMT > 1.4 mm), diabetes or uncontrolled hypertension were excluded. After the screening of consecutive 524 subjects according to the inclusion and exclusion criteria, a total of 402 subjects were finally enrolled. Written informed consent was obtained from participants and the ethics committee of PLA General Hospital approved present study.

### Demographic and clinical characteristics

Demographic and clinical characteristics such as age, gender, history of CVD, hypertension or current anti-hypertension therapy, and diabetic mellitus were obtained by the means of structured questionnaire. Blood pressures were measured by trained nurses using an automatic digital sphygmomanometer. Height, weight, waist circumference, hip circumference was measured manually. Body mass index (BMI) was calculated as weight (kilograms) divided by height (meters) squared.

### Laboratory examination

Fasting venous blood samples were collected from each participant and transferred to center laboratory in 30 minutes. Triglyceride (TG), TC, LDL-C, HDL-C, creatine kinase (CK), high sensitivity C-reactive protein (hs-CRP) and fasting blood glucose were measured by Automatic Biochemistry Analyzer (Roche, model c501, Sweden) after samples were centrifuged at 1500 g for 10 minutes. Glycosylated hemoglobin A_1c_(HbA_1c_) was measured in red blood cells using ion-exchange high-performance liquid chromatography (Sysmex, G8, Japan).

### Carotid ultrasonography

Carotid ultrasound examination was performed by the expert sonographers who were blinded to the participant’s clinical characteristics. Both the left and right carotid arteries were evaluated using high-resolution B-mode ultrasound scanners (Phillips, HD7, USA) equipped with a 7.5 MHz high-resolution linear array transducer (Phillips, L12-3, USA). The common carotid artery, carotid bulb, and internal carotid artery were carefully scanned from continuous angles to identify the thickest carotid IMT and plaque lesions. Intima-media thickness was defined as the distance between the leading edge of the lumen-intima and the leading edge of the media adventitia echo. Mean value of the three determinations was calculated and the final values of IMT were averaged by the left and right mean IMT values. Plaque was defined as a focal thickening of at least 50% greater than that of the surrounding vessel wall with a minimal thickness of at least 1.3 mm.

### Statistical analysis

Data are given as mean ± standard deviation (SD) or percentage. The Kolmogorov- Smirnov test was used to determine the normality of distributions and non-Gaussian distributed variables were log transformed (ie, TG). Linear correlations between carotid IMT (dependent variable) with lipid parameters and clinical characteristics (independent variable) were evaluated by Pearson correlation or Spearman rank correlation as appropriate. A stepwise multiple regression analysis was also performed to examine determinant factors for carotid IMT. The final model was determined using *P*_in_ < 0.05 and *P*_out_ < 0.10. Standardized coefficient (β) and *P* values are presented. The data were compared by the φ^2^-test or the Student’s t-test between the groups with and without carotid plaques. Next, multiple logistic regression models were constructed (*P*_in_ < 0.05 and *P*_out_ < 0.10) to elucidate the independent determinants of carotid plaque. The odds ratio and 95% confidence interval of each factor are given. Receiver operating characteristic (ROC) analysis was also performed to determine the ability of each lipid parameter to detect carotid plaque. Areas under the ROC curves (AUC) and 95% confidence intervals were calculated. All analyses were performed by SPSS statistical software (Version19.0, SPSS Inc). *P* values of <0.05 were considered statistically significant.

## Results

### Clinical characteristics

Baseline characteristics and clinical parameters of total subjects are shown in Table 1. The median age of participants was 50.16 years and about one-third (36.07%) of participants were female. Mean levels of total cholesterol, triglycerides, HDL-C, and LDL-C were 4.76 mmol/L, 1.86 mmol/L, 1.29 mmol/L and 3.10 mmol/L, respectively. Median levels of CK and hs-CRP were 110.62 U/L and 0.15 mg/dL, respectively. The prevalence of carotid plaque was 27.4%.

**Table 1:** **Clinical characteristics (n = 402)**

Variables	Mean ± SD or percentage
Gender, female (%)	145 (36.07)
Age (years)	50.16 ± 8.19
BMI (kg/m^2^)	25.40 ± 3.41
WHR	0.89 ± 0.08
SBP (mmHg)	124.04 ± 15.42
DBP (mmHg)	82.83 ± 11.44
Fasting blood glucose (mmol/L)	5.85 ± 1.10
HbA_1C_ (%)	5.90 ± 0.67
CK (U/L)	110.62 ± 71.24
Hs-CRP (mg/dL)	0.15 ± 0.12
TC (mmol/L)	4.76 ± 0.83
TG (mmol/L)	1.86 ± 1.49
HDL-C (mmol/L)	1.29 ± 0.33
LDL-C (mmol/L)	3.10 ± 0. 80
Carotid IMT (mm)	0.84 ± 0.20
Presence of carotid plaque (%)	110 (27.40)

*Abbreviations:* *BMI* body mass index, *WHR* waist-to-hip ratio *DBP* diastolic blood pressure, *SBP* systolic blood pressure, *TC* serum total cholesterol, *TG* serum triglycerides, *HDL-C* serum high-density lipoprotein cholesterol, *LDL-C* serum low-density lipoprotein cholesterol, *CK* creatine kinase, *Hs-CRP* high sensitivity C-reactive protein, *IMT* intima-media thickness.

### Correlation between carotid IMT and lipid parameters and other variables

In univariate correlation analysis (Table 2), carotid IMT was significantly correlated with age, LDL-C, non-LDL-C levels and LDL-C/HDL-C ratio (*p* < 0.001, *p* = 0.009, *p* = 0.008, *p* = 0.001). Carotid IMT was also correlated with gender, systolic blood pressure, HbA_1C_, CK, and total cholesterol levels (*p* = 0.043, *p* = 0.029, *p* = 0.024, *p* = 0.044, *p* = 0.017). However, there was no significant correlation between IMT and the other variables including triglycerides, HDL-C levels, TC/HDL-C ratio and hs-CRP levels.

**Table 2:** **Correlation between carotid IMT and lipid parameters and other variables**

Variables	Univariate		Multivariate	
	r	*p*-value	Standardized coefficient (β)	*p*-value
Gender	−0.110	0.043	NI	
Age	0.200	<0.001	0.193	<0.001
BMI WHR	0.009 −0.015	0.864 0.774	NI NI	
SBP	0.109	0.029	0.169	<0.001
DBP	0.001	0.990	NI	
Fasting blood glucose	0.018	0.730	NI	
HbA_1C_	0.119	0.024	NI	
CK	0.106	0.044	NI	
Hs-CRP	0.016	0.760	NI	
TC	0.126	0.017	NI	
TG	−0.018	0.736	NI	
HDL-C	−0.030	0.570	NI	
LDL-C	0.137	0.009	0.099	0.030
Non-HDL-C	0.140	0.008	NI	
TC/HDL-C ratio	0.087	0.099	NI	
LDL-C/HDL-C ratio	0.169	0.001	0.132	<0.001

Gender: Male = 1, female = 0.

*Abbreviation:* *NI* not included in the model.

Subsequently, stepwise multiple regression analysis was performed using 4 lipid parameters (TC, LDL-C, non-LDL-C, and LDL-C/HDL-C) and 5 other variables (gender, age, SBP, HbA_1C_, and CK) with a significance level <0.05 in previous simple correlation analysis as independent variables. As a result, age, systolic blood pressure, LDL-C and LDL-C/HDL-C ratio were finally included in the model as independent variables that interacted on carotid IMT (Table 2). LDL-C/HDL-C ratio was more strongly correlated with carotid IMT than LDL-C (β = 0.132, *p* < 0.001 vs. β = 0.099, *p* = 0.030). No association was seen between non-HDL-C and carotid IMT in the multivariate regression model.

### Associations between lipid parameters, other variables and the presence of carotid plaque

As shown in Table 3, age, systolic blood pressure, LDL-C and non-HDL-C levels, TC/HDL-C and LDL-C/HDL-C ratios were significantly higher, but HDL-C levels were significantly lower in subjects with carotid plaque than those without it. There was no significant difference between the two groups regarding the other clinical parameters such as percentage of female, BMI, WHR, HbA1c, CK, hs-CRP levels as well as the other lipid parameters such as total cholesterol and triglyceride levels.

**Table 3:** **Associations between lipid parameters, other variables and the presence of carotid plaque**

Variables	Plaque(+) (n = 110)	Plaque(−) (n = 292)	*p*-value
G/ender, female (%)	35 (31.82)	110 (37.67)	0.276
Age (years)	53.75 ± 7.67	47.46 ± 7.54	<0.001
BMI (kg/m^2^) WHR	25.49 ± 3.62 0.90 ± 0.08	25.34 ± 3.24 0.89 ± 0.08	0.743 0.205
SBP (mmHg)	126.46 ± 15.89	122.21 ± 14.84	0.029
DBP (mmHg)	83.66 ± 11.68	82.19 ± 11.26	0.310
Fasting blood glucose (mmol/L)	5.95 ± 1.10	5.76 ± 1.10	0.176
HbA_1C_ (%)	5.99 ± 0.67	5.84 ± 0.67	0.079
CK (U/L)	115.82 ± 88.99	106.70 ± 54.20	0.311
Hs-CRP (mg/dL)	0.16 ± 0.13	0.14 ± 0.12	0.092
TC (mmol/L)	4.87 ± 0.88	4.68 ± 0.78	0.070
TG (mmol/L)	1.96 ± 1.55	1.78 ± 1.45	0.324
HDL-C (mmol/L)	1.21 ± 0.28	1.35 ± 0.35	0.001
LDL-C (mmol/L)	3.22 ± 0. 85	3.01 ± 0. 76	0.042
Non-HDL-C (mmol/L)	3.65 ± 0.88	3.32 ± 0.76	0.002
TC/HDL-C ratio	4.19 ± 1.16	3.65 ± 1.00	<0.001
LDL-C/HDL-C ratio	2.74 ± 0.81	2.37 ± 0.80	<0.001
Mean IMT (mm)	0.84 ± 0.20	0.82 ± 0.16	0.410

Multiple logistic regression analysis was then performed using the presence of carotid plaque as the dependent variable, and using age, LDL-C and HDL-C levels as independent variables. The presence of carotid plaque was significantly and independently predicted by LDL-C (OR; 1.325, 95% CI; 1.046-1.821, *p* = 0.033) and HDL-C levels (OR; 0.093, 95% CI; 0.038-0.227, *p* < 0.001) after adjustment of age (Table 4).

**Table 4:** **Logistic regression model for prediction of the presence of carotid plaque**

Variables	Odds ratio	95% CI	*p*-value
Age	1.121	1.082-1.161	<0.001
HDL-C	0.093	0.038-0.227	<0.001
LDL-C	1.325	1.046-1.821	0.033

*Abbreviation:* *CI* confidence interval.

**Figure 1:**
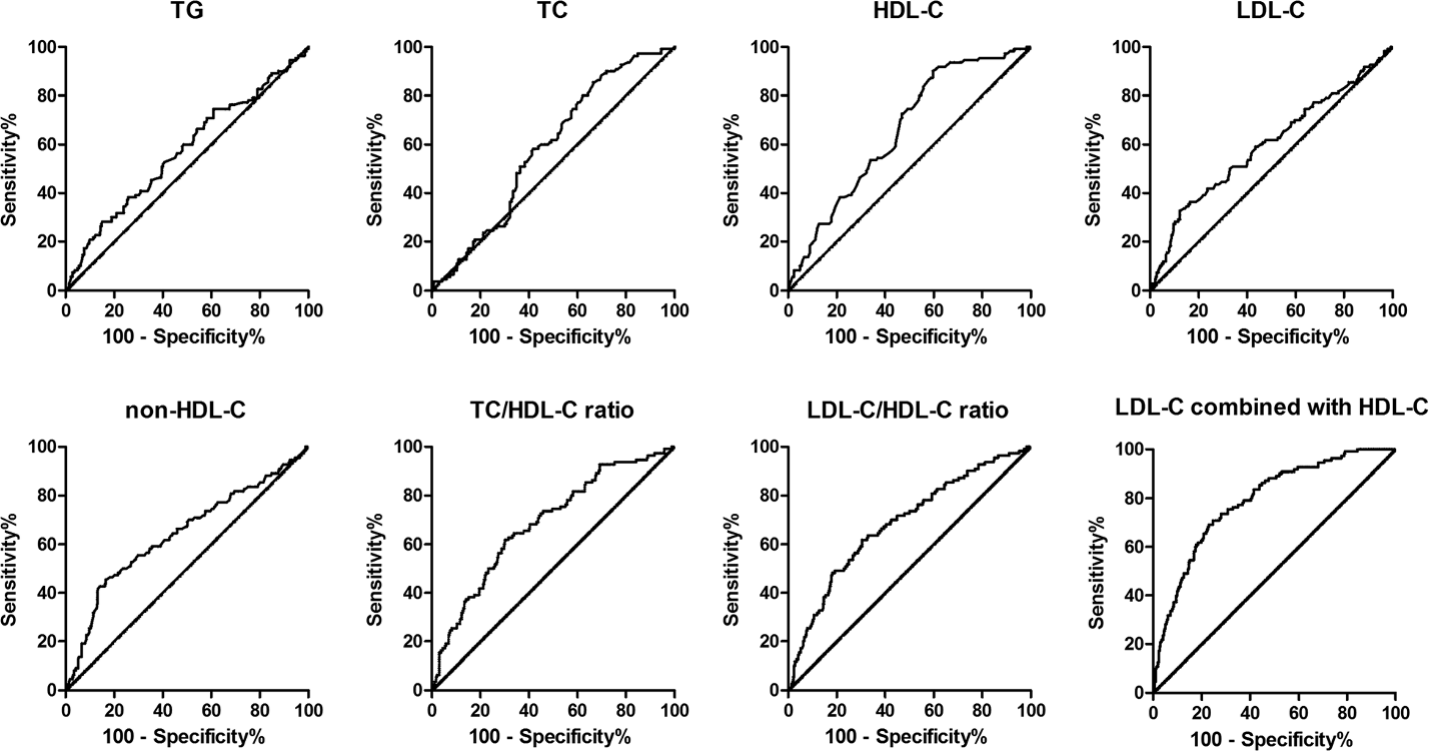
**ROC curves of lipid parameters and the combination model including LDL-C and HDL-C levels for predicting the presence of carotid plaque.**

Receiver operating characteristic analysis was also performed to test the accuracy of each of the 7 lipid parameters as well as the combination model including LDL-C and HDL-C levels for predicting the presence of carotid plaque. ROC curves are shown in Figure 1. The AUC-values for TC/HDL-C (0.685, 95% CI; 0.627–0.743, *p* < 0.001) and LDL-C/HDL-C ratios (0.683, 95% CI; 0.624–0.742, *p* < 0.001) were significantly higher than those for TC (0.565, 95% CI; 0.496–0.625, *p* = 0.037) and triglyceride levels (0.533, 95% CI; 0.496–0.570, *p* = 0.086), and relatively higher than those for HDL-C (0.657, 95% CI; 0.601–0.713, *p* < 0.001), non-HDL-C (0.641, 95% CI; 0.577–0.706, *p* < 0.001) and LDL-C levels (0.596, 95% CI; 0.531–0.662, *p* = 0.003). The combination model showed the largest area under the curve (0.788, 95% CI; 0.740–0.837, *p* < 0.001), which was significantly higher than the AUC-value of each lipid parameter separately used.

## Discussion

Although preventable, atherosclerotic cardiovascular disease remains a leading global cause of death and disability. Causal risk factors for cardiovascular disease constitute important therapeutic targets, but their usefulness as predictors for the disease developing is limited. Most heart attacks and strokes occur in people at average risk-factor level who are misclassified by traditional risk factor scoring, as low or intermediate risk [17]. Therefore, the only effective approach to restrict the health burden is to prevent the diseases from developing at the earliest possible stage of atherosclerosis. Thus, screenings for biomarkers for detecting early-stage asymptomatic vascular atherosclerotic changes are needed.

Our present study indicated that the combined application of LDL-C and HDL-C was superior to separate lipid parameters for assessing the risk of early-stage atherosclerosis in apparently healthy people from the general population. Carotid IMT appeared to be more closely correlated with LDL-C/HDL-C ratio compared with LDL-C level even after adjustment of age. Also, LDL-C/HDL-C ratio showed a positive association with the prevalence of carotid plaque. Furthermore, AUC-values for LDL-C/HDL-C and TC/HDL-C ratios were higher than those for the other lipid parameters and LDL-C combined with HDL-C level showed the highest area under the curve.

Low-density lipoprotein cholesterol transports cholesterol from the liver to peripheral tissues and advances the foaming of macrophages via uptake within the arterial wall. Conversely, high-density lipoprotein stimulates the efflux of excess cellular cholesterol and reversely transports it to the liver. High-density lipoprotein also protects against atherosclerosis by inhibiting cytokine-induced expression of endothelial cell adhesion molecules. According to these known mechanisms, it is no wonder that lipoprotein ratios, which indicate the proportion between the atherogenic and protective lipoproteins, have greater predictive power for assessing the extent of lipid accumulation in the arterial wall or the severity of atherosclerotic intimal changes.

Some studies have suggested that TC/HDL and LDL-C/HDL-C ratios are risk indicators for CVD with greater predictive value than each parameter used independently [10–14]. Furthermore, Katakami *et al*. [18] combined conventional lipid profile and lipid ratios to detect early-stage atherosclerosis in type 2 diabetic patients and noted that TC/HDL-C and LDL-C/HDL-C ratios can be good risk indicators for early-stage atherosclerosis, even if the conventional lipid parameters are within normal range. However, there were limit studies that investigated the effects of lipids and lipid ratios on early-stage atherosclerosis in general population. Kinosian *et al*. [14] reported that TC/HDL-C ratio or LDL-C/HDL-C ratio was superior to either TC level or LDL-C level for identifying general people at greater risk of developing subsequent coronary heart disease events. Tamada *et al*. [19] revealed that serum LDL-C/HDL-C ratio was independently associated with increased carotid plaque score and may represent a useful marker for evaluating the extent of atherosclerosis in the early stages in the general population compared with LDL-C alone. Coincidently, our results in multiple linear regression showed LDL-C/HDL-C ratio contributed to increased carotid IMT.

Recently, non-high-density lipoprotein cholesterol (non-HDL-C) has become increasingly recognized as an important index of atherogenic particles, as it reflects the cholesterol in all lipoprotein particles that are considered to be atherogenic. Non-HDL-C has been reported to be strongly associated with predicting coronary artery disease [6–9]. There also have been several reports about the association between non-HDL-C level and subclinical atherosclerosis in type 2 diabetic subjects [20–24]. Most of those studies noted that non-HDL-C level was a better predictor of carotid IMT than isolated lipid parameters, even TC/HDL-C ratio. However, according to our study, non-HDL-C failed to be an independent index for predicting early-stage atherosclerosis. The negative result may be attributable to the fact that our study population comprised numerous low-risk participants with relatively steady baseline non-HDL-C level than CVD patients. Further studies with a larger sample size are needed to avoid possible bias.

High sensitivity C-reactive protein (hs-CRP), a widely used marker of inflammation, was associated with neither carotid IMT nor presence of carotid plaque in this study. Hs-CRP has shown to be associated with cardiovascular disease events in most, but not all studies [25–27]. Our results indicate that routine use of hs-CRP in general people may not improve assessment for early-stage atherosclerosis, in accordance with Virani *et al.* [23].

The present study has several limitations. Firstly, this is a cross-sectional study with a relatively small number of subjects. Given the cross-sectional nature of our study, only associations can be drawn between LDL-C/HDL-C ratio and carotid early-stage atherosclerosis. But the associations do not necessarily indicate causality. Secondly, although we tried to minimize possible influence of known major confounders, the detailed information of smoking status (a known CVD risk factor) in study subjects were not available. The unmeasured factor may leave room for residual bias in multiple regression analysis. Moreover, the results may have been confounded by unknown factors. Thus, target LDL-C/HDL-C for management as a primary preventive strategy in early-stage atherosclerosis cannot be established from the present results. Further prospective cohort studies with a larger sample size are needed.

## Conclusions

Serum LDL-C/HDL-C ratio represents as an independent index associated with increased carotid IMT and LDL-C combined with HDL-C levels may be useful markers for predicting the presence of carotid plaque in the Chinese general population.
